# Microenvironmental Gene Expression Plasticity Among Individual *Drosophila melanogaster*

**DOI:** 10.1534/g3.116.035444

**Published:** 2016-10-20

**Authors:** Yanzhu Lin, Zhen-Xia Chen, Brian Oliver, Susan T. Harbison

**Affiliations:** * Laboratory of Systems Genetics, National Heart Lung and Blood Institute, National Institutes of Health, Bethesda, Maryland 20892; † Section of Developmental Genomics, Laboratory of Cellular and Developmental Biology, National Institute of Diabetes and Digestive and Kidney Diseases, National Institutes of Health, Bethesda, Maryland 20892

**Keywords:** microenvironmental plasticity, plasticity, *Drosophila*, interindividual variation, RNA-Seq

## Abstract

Differences in phenotype among genetically identical individuals exposed to the same environmental condition are often noted in genetic studies. Despite this commonplace observation, little is known about the causes of this variability, which has been termed microenvironmental plasticity. One possibility is that stochastic or technical sources of variance produce these differences. A second possibility is that this variation has a genetic component. We have explored gene expression robustness in the transcriptomes of 730 individual *Drosophila melanogaster* of 16 fixed genotypes, nine of which are infected with *Wolbachia*. Three replicates of flies were grown, controlling for food, day/night cycles, humidity, temperature, sex, mating status, social exposure, and circadian timing of RNA extraction. Despite the use of inbred genotypes, and carefully controlled experimental conditions, thousands of genes were differentially expressed, revealing a unique and dynamic transcriptional signature for each individual fly. We found that 23% of the transcriptome was differentially expressed among individuals, and that the variability in gene expression among individuals is influenced by genotype. This transcriptional variation originated from specific gene pathways, suggesting a plastic response to the microenvironment; but there was also evidence of gene expression differences due to stochastic fluctuations. These observations reveal previously unappreciated genetic sources of variability in gene expression among individuals, which has implications for complex trait genetics and precision medicine.

In many species, there are instances in which individuals of identical genotype have different phenotypes. Human diseases such as cancer (Lichtenstein *et al.* 2000), Type I and Type II diabetes (Kaprio *et al.* 1992; Hyttinen *et al.* 2003), multiple sclerosis (Willer *et al.* 2003), Alzheimer’s disease (Pedersen *et al.* 2004), Parkinson’s disease (Tanner *et al.* 1999), narcolepsy (Pollmacher *et al.* 1990), and insomnia (Watson *et al.* 2006) can be discordant between a monozygotic twin and its sibling. This discordance among genetically identical individuals is an example of phenotypic plasticity, which is a change of phenotype in response to environmental changes (Callahan *et al.* 1997). For some diseases, evidence implicates environmental factors unique to each individual in a twin pair, rather than environmental factors common to both twins, as a possible source of the discordance (Kaprio *et al.* 1992; Lichtenstein *et al.* 2000; Hyttinen *et al.* 2003; Watson *et al.* 2006). Environmental factors unique to an individual within an otherwise common environment can be defined as microenvironmental effects (Hill and Mulder 2010). Breeding programs involving economically important animals and plants have reported that microenvironmental effects are under partial genetic control. Genetic variation in the microenvironmental variability of a wide variety of traits has been documented, such as litter size in sheep, pigs, mice, and rabbits (SanCristobal-Gaudy *et al.* 2001; Sorensen and Waagepetersen 2003; Gutierrez *et al.* 2006; Ibanez-Escriche *et al.* 2008b; Yang *et al.* 2011); body weight measures in snails, chickens, mice, and rabbits (Ros *et al.* 2004; Gutierrez *et al.* 2006; Rowe *et al.* 2006; Garreau *et al.* 2008; Ibanez-Escriche *et al.* 2008a); and grain yield in maize (Yang *et al.* 2012). Furthermore, studies have demonstrated that gene expression variability among humans has a genetic basis (Li *et al.* 2010; Mar *et al.* 2011; Hulse and Cai 2013). One difficulty with outbred populations is that nonadditive genetic variance and environmental variance are confounded, making computation of the relative contributions of either component difficult (Hill and Mulder 2010). In contrast, variability in phenotypes among genetically identical individuals within a common environment can be considered a measure of phenotypic plasticity in response to microenvironmental changes (Morgante *et al.* 2015). Recent work has therefore employed inbred (or isogenic) lines in model organisms in order to measure microenvironmental plasticity directly, and to understand its underlying genetic basis. Microenvironmental plasticity in phenotypes is common among inbred model organisms. For example, individual plants exposed to the same environmental conditions respond differently in their morphology and fitness traits within recombinant inbred lines of *Arabidopsis thaliana* (Hall *et al.* 2007; Sangster *et al.* 2008) and maize (Ordas *et al.* 2008). Moreover, isogenic strains of yeast exhibit variability in gene expression (Blake *et al.* 2006; Ansel *et al.* 2008), and studies using *Drosophila melanogaster* have identified phenotypic differences among genetically identical flies for wing shape (Whitlock and Fowler 1999); for sternopleural and abdominal bristle number (Mackay and Lyman 2005); for sleep and activity measures (Harbison *et al.* 2013); for chill coma, startle response, and starvation resistance (Morgante *et al.* 2015); for locomotor handedness (Ayroles *et al.* 2015); and for food intake (Garlapow *et al.* 2015). Genomic polymorphisms associated with within-genotype variability and microenvironmental plasticity have been identified for some traits (Mackay and Lyman 2005; Hall *et al.* 2007; Ansel *et al.* 2008; Ordas *et al.* 2008; Sangster *et al.* 2008; Harbison *et al.* 2013; Ayroles *et al.* 2015; Garlapow *et al.* 2015; Morgante *et al.* 2015), but they do not indicate how phenotypic differences between individuals of identical genotype in common environmental conditions might arise. One possibility is that within-genotype, within-environment differences develop from individualized gene expression profiles.

Several studies support the notion that differences in gene expression explain microenvironmental phenotypic plasticity. Gene expression has been measured in lymphoblastoid cell lines derived from monozygotic twins discordant for rheumatoid arthritis (Haas *et al.* 2006), schizophrenia (Kakiuchi *et al.* 2008), and bipolar disorder (Kakiuchi *et al.* 2003; Matigian *et al.* 2007); from monocytes and skin fibroblasts of monozygotic twins discordant for Type I diabetes (Beyan *et al.* 2010; Caramori *et al.* 2012); and from fat cells of monozygotic twins discordant for obesity (Pietilainen *et al.* 2008). Differences in the expression of key genes and genetic pathways relevant to disease were found between discordant twins. Further, studies measuring chromatin marks, which can activate or repress transcription, report that despite common environmental influences, differences in the epigenetic milieu are present in young monozygotic twins, and they only increase over time (Fraga *et al.* 2005). In addition, an experiment examining the trade-off between pooling RNA samples to save costs and the ability to identify differentially expressed genes in rats revealed that pooling samples may obscure the variation in gene expression among individuals (Kendziorski *et al.* 2005). This work implies that fluctuations in gene expression have the capacity to alter phenotypes in genetically identical individuals, and may contribute to the etiology of disease. Two difficulties with these studies is the lack of control over environmental exposures, and, of course, within-genotype sample size is limited in twin studies. This is not an issue for *Drosophila* experiments, in which identical genotypes can be grown in abundance under restricted environmental conditions.

At present, the number of transcripts, and the extent to which they differ in abundance among genetically identical metazoans reared in identical environmental conditions, is not known. Is the transcriptome robust to microenvironmental perturbations, or is it plastic (Gibson 2008)? Are expression differences among genetically identical individuals heritable? Differences in gene expression among genetically identical individuals, if present, could be due to individual responses to microenvironmental perturbations, or they could be due to stochastic factors originating at the cellular level. To answer these questions, we measured gene expression in individual flies in a highly replicated and environmentally controlled study. Our objectives were to determine whether there are gene expression differences among identical individuals in a common environment, and to determine whether these gene expression differences originate from defined biological responses to the environment, indicating a genetic origin, or whether they reflect stochastic cellular processes. To fulfill these objectives, we sequenced mRNA from individual flies from 16 *Drosophila* Genetic Reference Panel (DGRP) genotypes, which are inbred lines derived from wild-caught flies, nine of which are infected with *Wolbachia* (Mackay *et al.* 2012; Huang *et al.* 2014). We performed RNA-Seq on three biological replicates of the experiment while maintaining the same environmental conditions. We used eight flies for each genotype, environment, and sex condition, which after quality control procedures resulted in sequence data for 730 individual flies.

Our analyses reveal that gene expression exhibits microenvironmental plasticity. Analysis of mean gene expression suggested that 23% of the transcriptome fluctuates among individual flies of identical genotype within a common environment, that the transcripts fluctuating among individuals were moderately heritable, and that the transcripts originated from specific biological processes rather than stochastic or technical effects. We quantified microenvironmental plasticity in gene expression as the variation in gene expression among individuals within genotype, replicate, and sex, and found that it was heritable for 7.3% of the transcriptome. Microenvironmental plasticity in gene expression did not always map to specific biological processes, had low heritability, and may be partially stochastic in origin. Thus, certain categories of genes respond to microenvironmental perturbations, while others are quite robust. These findings reinforce the need to consider the influence of environmental plasticity on the genetic basis of complex traits and disease; the analysis of a trait is relatively straightforward if it is influenced by genes with robust expression across individuals, but far more challenging if it is influenced by genes that are plastic.

## Materials and Methods

### Drosophila lines and culturing

We chose the following 16 lines of the DGRP (Mackay *et al.* 2012; Huang *et al.* 2014) at random for this experiment, after excluding five slow-growing lines: *DGRP-93*, *DGRP-229*, *DGRP-320*, *DGRP-352*, *DGRP-370*, *DGRP-563*, *DGRP-630*, *DGRP-703*, *DGRP-761*, *DGRP-787*, *DGRP-790*, *DGRP-804*, *DGRP-812*, *DGRP-822*, *DGRP-850*, and *DGRP-900*. We maintained strictly controlled replicate environments by using the following procedure. We seeded cultures with five males and five females of each line on standard *Drosophila* medium (http://flystocks.bio.indiana.edu/Fly_Work/media-recipes/bloomfood.htm) in numerical genotype order on a single shelf in one incubator maintained at 25°, 60% humidity, and a 12:12-hr light:dark cycle. We collected virgin males and females from the parental cultures, and maintained them at 20 to a same-sex vial for 4 d at the same location in the incubator to control for potential effects of mating status (Isaac *et al.* 2010) and social exposure (Ganguly-Fitzgerald *et al.* 2006). Three separate biological replicates of the experiment were performed. At the end of the 4-d period, eight flies of each genotype/sex/replicate were anesthetized and frozen on dry ice beginning at 1:00 pm in randomized genotype order in Axygen 96 Deep-well plates (Corning, Corning, NY) containing 200 μl of 1.0 mm glass beads. Replicate environments were stratified across plates. Additional details concerning our experimental approach can be found in (Lin *et al.* 2016) and in the Gene Expression Omnibus (GEO) entry (GSE60314) (see “DGRP_Number,” “Sex,” Environment,” “Fly_Number,” and “Fly_Plate_Location” in the GSE60314_GEO_run_summary.xlsx file, GEO entry GSE60314, for the order of genotypes, sexes, and replicate environments).

### Total RNA extraction, mRNA isolation, library preparation, and sequencing

We extracted the total RNA of each fly using an RNeasy 96 Plate kit (Qiagen, Valencia, CA). We then added 96 ERCC spike-in standards (External RNA Controls Consortium, SRM2374, beta version, pools 78A/78B) to each total RNA sample before proceeding with library preparation. We prepared 300–350 bp stranded PolyA libraries for each fly following the method of Wang *et al.* (2011), with modifications to the procedure as detailed in Lin *et al.* (2016) in a 96-well plate format. Using equal amounts of each library, we pooled libraries in groups of 24 for sequencing in plate order (see the “Sequence_Run_ID” for the 4-letter multiplex pool identifier in the GSE60314_GEO_run_summary.xlsx file, GEO entry GSE60314). Each library in the pool had a unique index, or “bar code”, sequence added. We sequenced the libraries on a HiSeq2000 (Illumina, San Diego, CA) in 76-bp, single-end sequencing reactions. We mapped those reads that passed Chastity base-calling filters (score > 0.6) (CASAVA 1.8.2, Illumina, San Diego, CA).

### Sequence mapping and alignment

We mapped sequence reads to release 6 version 01 of the *D. melanogaster* reference genome (FlyBase file: dmel-all-chromosome-r6.01.fasta), with sequence-corrected ERCC sequences [ERCC_reference.fa, see (Jiang *et al.* 2011)] added. We used TopHat2 (v2.0.10) with nondefault parameters “-g 1–library-type fr-firststrand” (Kim *et al.* 2013). We mapped pre-miRNAs, pseudogenes, mRNAs, ncRNAs, rRNAs, snoRNAs, snRNAs, tRNAs, genes, coding sequences, and exons. We used HTSeq (Anders *et al.* 2015) to count the number of reads per gene using the option “–stranded=reverse -i gene_id -t exon” (Lin *et al.* 2016).

### Sequence quality control

We assessed the quality of the sequence data with a series of checking procedures; the methods and calculations used are provided in greater detail in Lin *et al.* (2016), and in the GEO entry (GSE60314). Briefly, we first verified that the sequence data for each fly had the expected index or bar code, and kept data for flies with 95% or greater of the expected index in the analysis. We selected 118 flies at random, and prepared duplicate libraries for them (Lin *et al.* 2016). We verified that the technical variance due to library preparation was low as compared to the biological variance (Lin *et al.* 2016). As the DGRP is fully sequenced (Mackay *et al.* 2012; Huang *et al.* 2014), we compared the published sequence of each line to each fly sequence in order to verify the genotype of each fly. We required our sequence data to map back to the expected DGRP genotype with 5% or less mismatch (Lin *et al.* 2016). We also verified the sex of each fly by correlating each fly’s sequence with a reference standard of the same sex, and contrasting it with the opposite sex (Lin *et al.* 2016). These checks resulted in the retention of sequence data for 730 samples. Additional information can be found in the summary table in the GEO entry GSE60314 (GSE60314_GEO_run_summary.txt). The table includes information concerning the location of each fly in each 96-well plate; RNA quantities; library plate locations and quantities; the index sequences used to identify the sequence of each individual fly in each sequence pool, sequence run parameters, machine, lane, and flow cell IDs, and numbers of mapped reads.

### Differential gene expression analysis and clustering

Out of 17,023 annotated genes in the *D. melanogaster* genome, we had read counts above zero for 16,623 genes. The remaining 400 annotated genes had zero read counts for all flies, so we eliminated these genes from each dataset. We normalized the read count data using the DESeq normalization method (Anders and Huber 2010). We derived an empirical low expression threshold, 3.486 DESeq-normalized read counts, from the overlap of the distribution of intergenic and genic read counts as detailed in Lin *et al.* (2016). We eliminated genes from the dataset that had read counts below this threshold for all flies. This ended up being 949 genes, leaving 15,674 expressed genes. We used Levene’s test, and the Brown-Forsythe test, to calculate the heterogeneity of variance across genotype, sex, and replicate. The results of these tests suggested extensive heterogeneity of variance. To determine which genes were differentially expressed across the main factors of Genotype, Sex, and Replicate, and their interactions, we assumed that the number of read counts for gene *i* in sample *k* can be modeled by a negative binomial distribution, where

$$x_{\mathrm{ik}}∼\text{NB}\left({µ}_{\mathrm{ik}}\text{,}\sigma_{\mathrm{ik}}^{\text{2}}\right)$$

where *µ_ik_* is the mean and *σ_ik_*^2^ is the variance. The mean is given by

$$E\left(x_{\mathrm{ik}}\right)=\mu_{\mathrm{ik}}$$

and the variance can be written in terms of the mean:

$$\sigma_{\mathrm{ik}}^{\text{2}}=\mu_{\mathrm{ik}}+\phi \mu_{\mathrm{ik}}^{\text{2}}$$

where *ϕ* is a dispersion parameter that indicates how much the variance exceeds the mean (Anders and Huber 2010).

We then tested each gene for differential gene expression across the main factors of Genotype, Sex, and Replicate, and their interactions, using the following generalized linear model, assuming a negative binomial distribution:

$$\log\left(\mu_{\mathrm{ik}}\right)=\beta_{0}+S+G+R+G\times R+S\times G+S\times R+S\times G\times R$$

where *S* is sex, *G* is DGRP genotype, and *R* is the replicate environment. We tested each term in the model using a full and a reduced model as shown below:

$$\text{Model}\;\text{1:}\log\left(\mu_{\mathrm{ik}}\right)=\beta_{\text{0}}+S+G+R$$

$$\text{Model}\;\text{2}\left(\text{a}\right)\text{:}\log\left(\mu_{\mathrm{ik}}\right)=\beta_{\text{0}}+S+G+R+G\times R$$

$$\text{Model}\;\text{2}\left(\text{b}\right)\text{:}\log\left(\mu_{\mathrm{ik}}\right)=\beta_{\text{0}}+S+G+R+G\times R+G\times S$$

$$\text{Model}\;\text{2:}\log\left(\mu_{\mathrm{ik}}\right)=\beta_{\text{0}}+S+G+R+G\times R+G\times S+R\times S$$

To test the main effect of sex, genotype, and replicate, we used Model 1 as the full model, and calculated the likelihood ratio between Model 1 and a reduced Model 1 with each of the main effects removed in turn. We tested the two-way interaction terms *G* × *R*, *G* × *S*, and *R* × *S* using the same approach. We applied Model 2(b) and 2(a) to find genes with a significant *G* × *S* interaction, for example; Model 2(b) was the full model, while Model 2(a) was the reduced model. To test the significance of the three-way interaction term *S* × *G* × *R*, we again used the same approach, where Model 2 was the reduced model. The likelihood ratio statistic comparing any of the two models is simply the difference between the deviances of the full model and the reduced model. We implemented this analysis using the DESeq package (Anders and Huber 2010), and limma-voom, using the “voomWithQualityWeights” function to account for heteroscedasticity across samples (Law *et al.* 2014). We applied a false-discovery rate (FDR) threshold of < 0.05 to correct for multiple tests (Benjamini and Hochberg 1995). The overlap of differentially expressed genes between the two programs was high, and the numbers of differentially expressed genes were very large using limma-voom; thus, we chose the DESeq analysis as it was more conservative. Finally, in order to determine whether there was any confounding between genotype and potential sources of technical variation, such as plate and multiplex library pool, we analyzed a model that considered these effects as covariates: log(*μ_ik_*) = *β_0_* + Plate + Pool + *S* + *G* + *R* + *G* × *R* + *S* × *G* + *S* × *R* + *S* × *G* × *R*. We calculated the microenvironmental plasticity of gene expression as the coefficient of environmental variation (CV_E_) using the equation 100*σ/*µ* where *σ* is the SD of DESeq-normalized read counts within each gene for each genotype/sex/replicate, and *μ* is the mean (Mackay and Lyman 2005; Morgante *et al.* 2015); this measure has previously been used to represent variation in gene expression (Ansel *et al.* 2008). We analyzed the resulting expression CV_E_ as a trait using the following ANOVA model: *Y* = *μ* + *S* + *G* + *G* × *S* + *ε*, where *S* and *G* are as defined above, and *ε* is error. As the model was applied to each gene separately, we used an FDR threshold of < 0.05 to correct for multiple testing. We used Modulated Modularity Clustering (MMC) (Stone and Ayroles 2009) to cluster significantly differentially expressed genes. We used Gene Ontology (GO) (Ashburner *et al.* 2000; Thomas *et al.* 2003) to examine the clusters for significant biological process categories. Gene lists were compared using the entire *Drosophila* genome as a background. Enrichment of genes within a GO category was deemed significant if the Bonferroni-adjusted *P*-value was < 0.05. We estimated broad-sense heritability (*H*^2^) for gene expression as *H*^2^ = (*σ_G_* + *σ_GR_* + *σ_GS_* + *σ_GRS_*)/(*σ_G_* + *σ_GR_* + *σ_GS_* + *σ_GRS_* + *σ_ε_*), where *σ_G_* is the variance component among genotypes, *σ_GR_* is the genotype-by-replicate variance component, *σ_GS_* is the genotype-by-sex variance component, *σ_GRS_* is the genotype-by-replicate-by-sex variance component, and *σ_ε_* is the sum of all other sources of variation. *H*^2^ for gene expression CV_E_ was estimated as *H*^2^ = (*σ_G_* + *σ_GS_*)/(*σ_G_* + *σ_GS_* + *σ_ε_*). We estimated the effects of *Wolbachia pipientis* infection by classifying each DGRP line as infected or uninfected as previously published (Huang *et al.* 2014), and calculating the following general linearized model for each gene: log(*µ*_ik_) = *β*_0_ + *I*, where *I* is infection status. Additionally, for each gene, we correlated the SD in gene expression per line with reported percentages of residual heterozygosity per line in the DGRP (Huang *et al.* 2014), and with total rRNA levels using the Spearman correlation method.

### Data availability

All RNA-Seq data from this study are available from the National Center for Biotechnology Information (NCBI) GEO under the accession number GSE60314.

## Results

**Figure 1:**
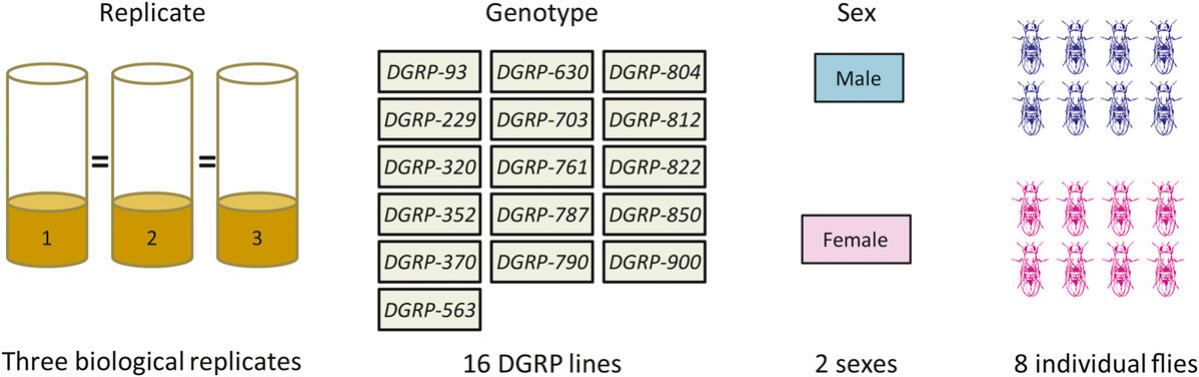
Experimental design.

We sequenced poly-A selected RNA from 768 individual flies in order to systematically explore the relationship between magnitude and variability in gene expression among individuals (Figure 1). We used flies from 16 DGRP lines chosen at random for the study. Flies of each genotype were cultured as three biological replicates; the environmental conditions for each replicate were carefully controlled and designed to be equivalent (*Materials and Methods*). In order to explore gene expression differences among individual flies, we sequenced eight males and eight females for each DGRP line/replicate condition. After applying our quality control standards to the data (Lin *et al.* 2016), sequences from 730 flies remained. We used a generalized linear model to examine differences among genotypes, replicates, sexes, and their interactions, using read counts per gene as a proxy for gene expression [(Lin *et al.* 2016) and *Materials and Methods*]. Large numbers of genes were differentially expressed among experimental factors using the DESeq analysis (Table 1; Supplemental Material, Table S1), even at very low FDRs (Table 1 and Table S2). The limma-voom analysis identified very large numbers of differentially expressed genes for each factor at an FDR of 0.05. The overlap was relatively high between the two methods, ranging from 62.8% for the main effect of replicate environment to 99.6% for the interaction effect of genotype, replicate, and sex (Table S3). The addition of plate and multiplex library pool effects to the model did not appreciably affect genes identified as differentially expressed among genotypes, though it did affect genes differentially expressed in replicate environments; differentially expressed genes for factors containing genotype overlapped by ≥ 92% (Table S4). Below, we address the relationship between the mean and variance of gene expression among each of the experimental factors (genotype, replicate, sex, and their interactions) in turn.

**Table 1:** Numbers of genes with differential expression and differential CV_E_

Factor	Differentially Expressed Genes*^a^**^,^**^b^*	Genes with Differential *CV*_E_*^b^**^,^**^c^*	Overlap
*Genotype* (*G*)	10,401(66.4)	1212(7.3)	1135
*Replicate* (*R*)	9607(61.3)	N/A	N/A
*Sex* (*S*)	14,883(94.9)	13,225(79.6)	12,738
*G* × *R*	3463(22.1)	N/A	N/A
*G* × *S*	7148(45.6)	266(1.6)	246
*R* × *S*	2646(16.9)	N/A	N/A
*G* × *R* × *S*	3654(23.1)	N/A	N/A

a Numbers of genes that were significantly differentially expressed at an FDR < 0.05.

b Numbers in parentheses indicate the percentage of the total number of expressed genes (15,674).

c Numbers of genes with differential CV_E_ at an FDR < 0.05.

### Gene expression differences among individual flies

**Figure 2:**
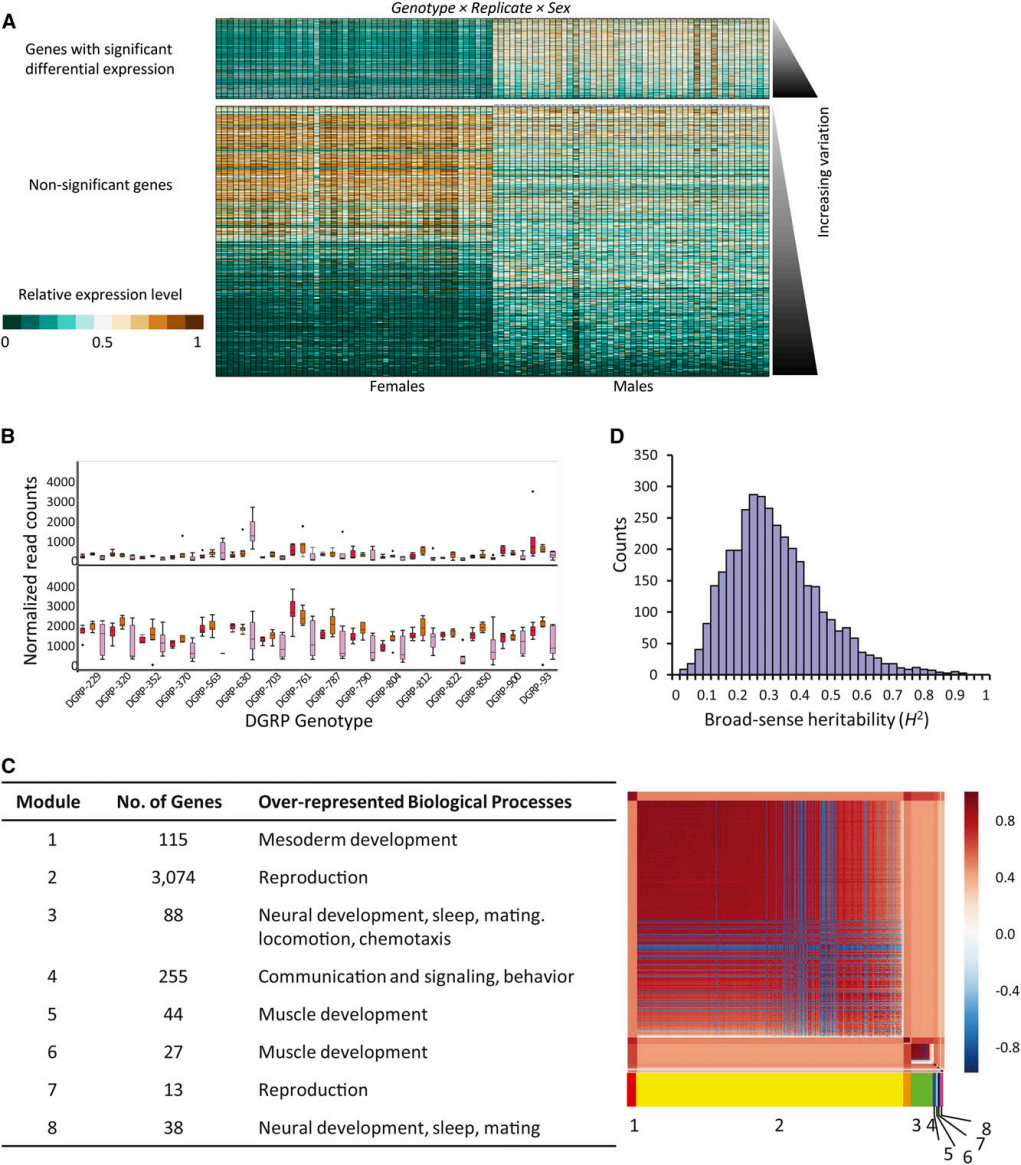
Genes differentially expressed among individual flies. (A) Heat map showing the relative expression levels (DESeq-normalized read counts/maximum DESeq-normalized read counts) of every detected gene in the genome, by genotype, replicate environment, and sex. The top section of the plot shows the genes with significant differential expression, while the bottom shows the nonsignificant genes. The genes are ordered from top to bottom by increasing variability as measured by dispersion (*Materials and Methods*). Females are plotted on the left-hand side; males are plotted on the right. Genotypes are ordered numerically with each replicate (*i.e.*, *DGRP-229* Replicate 1, *DGRP-229* Replicate 2, and *DGRP-229* Replicate 3). (B) Representative box plot showing the variation among genotype, replicate, and sex in *ninaC*. Red boxes indicate Replicate 1; orange boxes indicate Replicate 2; and pink boxes indicate Replicate 3. (C) MMC of genes differentially expressed among individual flies. The red-white-blue color scale indicates the correlation among genes. Clusters of genes (modules) are ordered from the highest connectivity on the upper left to the lowest connectivity on the lower right. The extent of each module is marked with a color bar. The table to the side lists some of the biological processes over-represented in each module; for the complete list of biological processes, see Table S9. (D) Distribution of broad-sense heritability (*H*^2^) for genes differentially expressed among individuals.

Flies of identical genotype and sex grown under replicated conditions should show the lowest differential expression. However, differences in gene expression were common. Three lines of evidence suggested that differences in gene expression in individual flies were present. First, 3654 of 15,674 detected transcripts were differentially expressed across genotype, replicate, and sex (Table 1 and Table S1). This observation implied that replicates of identical genotype and sex had mean gene expression differences. Second, 3415 of these 3654 genes had significant heterogeneity of variance within genotype, replicate, and sex by Brown-Forsythe’s test (FDR < 0.05), and 3610 had significant heterogeneity of variance by Levene’s test (FDR < 0.05) (Table S5). We inferred from these results that differences in gene expression among individuals within a genotype/replicate/sex condition were present. Third, we previously demonstrated that differences in gene expression between duplicate libraries prepared for 118 of the flies used in this study originated from biological differences, and the differences due to technical factors were small (Lin *et al.* 2016). Thus, individual flies of identical genotype reared and maintained in a common environment had differential gene expression, and the source of these differences was biological, not technical, variation. Gene expression differences among individuals tended to be male-biased (Figure 2A); 3254 genes (89.1%) showed male-biased expression, in contrast to 6967 (61.8%) of the remaining genes in the transcriptome with male-biased expression (Fisher’s exact test, *P* < 0.0001). A representative example is shown for *ninaC*, a gene with functions in phototransduction (Montell and Rubin 1988) that may be downstream of *dsx* in the sex differentiation pathway (Goldman and Arbeitman 2007). Clear differences in the expression of this gene in individuals of identical genotype, sex, and replicate can be seen in the sizes of the box plots (Figure 2B).

A small fraction of genes differentially expressed among individuals, 68 genes in total, may have originated from differential responses to *W. pipientis* infection known to segregate among lines of the DGRP (Huang *et al.* 2014). Among these 68 genes were three amylase genes (*Amy-d*, *Amy-p*, and *Amyrel*), but we observed no additional functional patterns (Table S6). Furthermore, lines of the DGRP were developed by inbreeding wild-caught flies; this manipulation makes most of the genome homozygous, but some regions will remain heterozygous within a line. Heterozygosity in the line could segregate into three separate genotypes among individual flies, which could in turn result in gene expression differences among individuals. We therefore correlated the SD of expression per line with the percentage of heterozygosity in the DGRP that remained after the inbreeding procedure (Huang *et al.* 2014), with the null hypothesis that the correlations are zero as the flies are largely homozygous. We found that 133 genes were significantly correlated with heterozygosity (Table S7). In addition, we noted 89 histone-encoding genes, which are not polyadenylated (Marzluff 2005) and two nonprotein-coding genes that were correlated with total rRNA levels (Table S8); individual expression differences in these two classes of genes are unlikely to have biological meaning, as they track polyA+ isolation efficacy. Thus, known historical pathogen exposure and genomic sequence features of the DGRP could explain part, but far from all, of the individual differences in gene expression that we observed. Like *ninaC*, the bulk of the genes showing differential expression among individual flies had functions in physiological responses to the environment or roles in behavior, as detailed below.

If gene expression is plastic, that is, it is responsive to environmental perturbations (Debat and David 2001), then the genes that differ among individuals should encode coherent functions operating in pathways that interact with the environment. On the other hand, if the differences in gene expression among individuals are stochastic fluctuations, then these genes will not cluster into known relationships. We looked for relationships among the genes differentially expressed among individuals using MMC (Stone and Ayroles 2009) coupled with GO (Ashburner *et al.* 2000; Thomas *et al.* 2003) analysis to identify groups (or modules) of genes with over-represented biological process categories (Bonferroni-corrected *P* < 0.05). The genes clustered into eight separate modules with diverse roles (Figure 2C, Table S8, and Table S9). Module 1 was enriched for genes involved in mesoderm development, including the development of muscles and reproductive organs; Modules 5 and 6 were also enriched for genes implicated in muscle development. Most of the genes (3074) clustered into Module 2, which, along with Module 7, was enriched for genes with functions in reproduction, including female receptivity, copulation, and insemination. Genes with reproduction-related functions in Module 2 included those encoding 17 accessory gland proteins/peptides, two chorion proteins, all three ejaculatory bulb proteins, five male sterile genes, 15 male-specific transcripts, 22 seminal fluid proteins, four vitelline membrane proteins, *Mst89B*, *ovo*, *quick-to-court*, and *Sex Peptide*. Modules 3 and 8 were enriched for genes implicated in synaptic transmission, ion transport, and neural development, as well as genes involved in regulating mating, locomotion, sleep, and chemotaxis behaviors. Examples include *bruchpilot*, *couch potato*, *ether a go-go*, *fruitless*, *Hyperkinetic*, *Resistant to dieldrin*, *Shaker*, and *slowpoke*. Module 4 was enriched for cell communication and signaling, exocytosis, and the response to light and other stimuli. Broad-sense heritability (*H*^2^) measures how much variation in gene expression is due to genotype. *H^2^* estimates for genes differentially expressed among individuals ranged from 0 to 0.912 with a mean of 0.310 ± 0.15 SD, indicating that there is a genetic basis for at least some of the individualized expression profiles (Figure 2D and Table S1). The predicted coregulated networks (Harbison *et al.* 2009) that we identified implicate physiological processes and behaviors interacting with the environment, and suggest that differences in gene expression among individual flies are more likely related to extrinsic regulatory factors rather than intrinsic stochastic events such as transcriptional noise.

### Microenvironmental plasticity in gene expression

**Figure 3:**
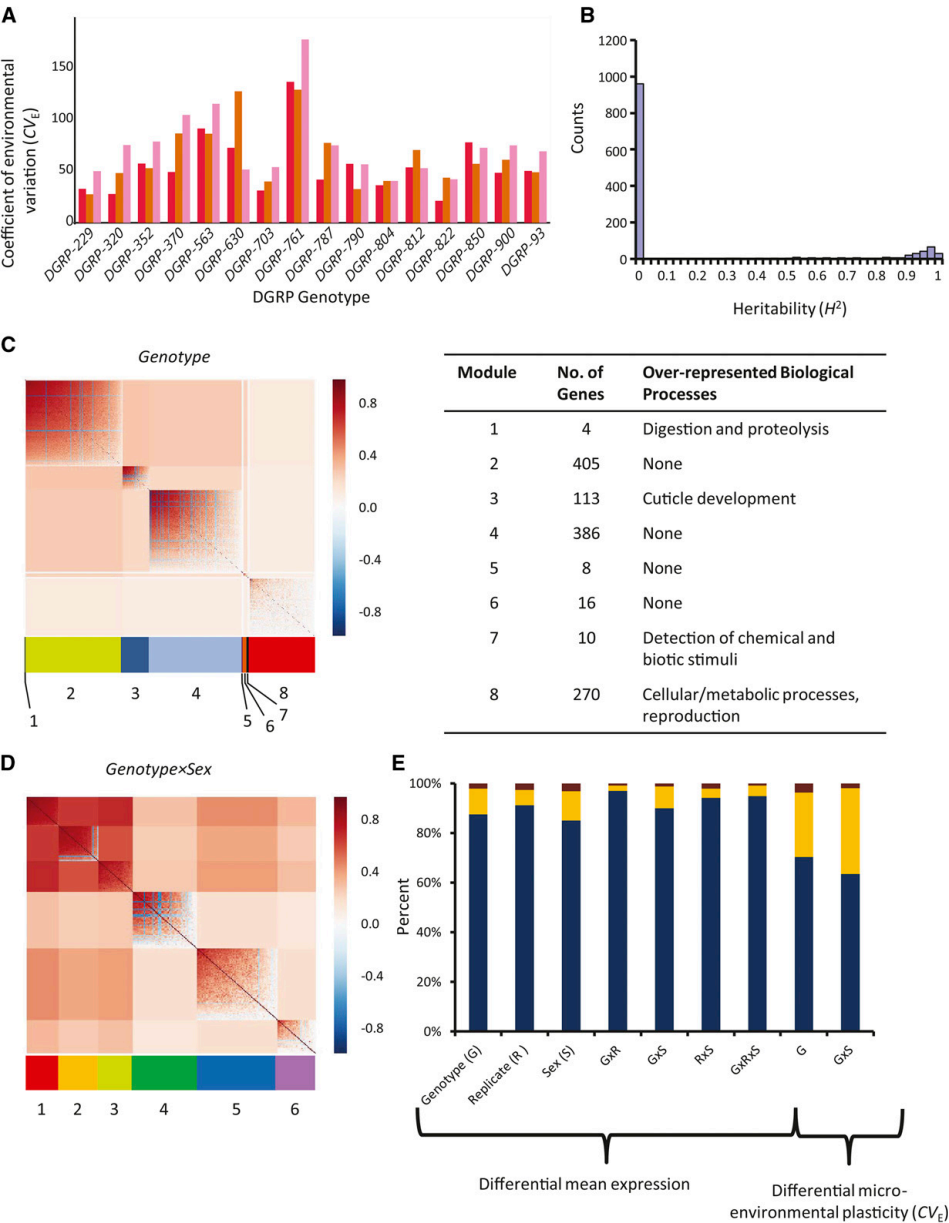
Microenvironmental plasticity genes. (A) Representative bar graph of the CV_E_ for *Cytochrome P450-6a8* across genotypes. The colors of the bars indicate the replicates as in Figure 2. (B) Distribution of broad-sense heritability for transcripts exhibiting genetic variation in microenvironmental plasticity. (C, D) MMC modules of genes differentially variable among (C) genotypes and (D) genotype-by-sex. The color scale and cluster orientation is the same as in Figure 2. (E) Bar graph showing the percentage of protein-coding *vs.* nonprotein-coding genes for genes with significant mean differential expression, and those with differential microenvironmental plasticity (CV_E_). Blue, protein-coding genes. Yellow, nonprotein-coding genes. Red, other/unknown.

We next asked whether the gene expression differences among individual flies were genetically based or stochastic. We used the coefficient of environmental variation (CV_E_, *Materials and Methods*), to quantify the expression differences among individuals as the microenvironmental plasticity in gene expression (Mackay and Lyman 2005; Morgante *et al.* 2015). A total of 1212 genes had CV_E_ values that varied significantly with DGRP genotype, indicating that microenvironmental plasticity in gene expression has a genetic basis (Figure 3A, Table 1, and Table S10; see Figure S2A for the relationship between dispersion across genotypes irrespective of mean expression), while 264 genes had genetic differences in CV_E_ that were sex-specific. Differential CV_E_ values between males and females were also observed (Figure S1C and Figure S2B). Thus, microenvironmental plasticity in gene expression depended upon underlying genotype and on sex.

Broad-sense *H*^2^ estimates of CV_E_ ranged from 0 to 0.988, with a mean of 0.177 ± 0.36 SD. Unlike the moderate heritabilities in gene expression observed for genes differentially expressed among individuals (Figure 2D), heritabilities for microenvironmental plasticity were often near zero; 1004 genes had very low *H*^2^ (contrast Figure 2D with Figure 3B). The 258 genes with high *H*^2^ included immune response genes (*Attacin-D*, *Cecropin C*, *Defensin*), genes encoding cuticular proteins, *doublesex-Mab related 93B*, and *doublesex cognate 73A*. To explore overall patterns of function, the genes were clustered into genotype and genotype-by-sex modules (Figure 3, C and D, Table S11, and Table S12). Microenvironmental plasticity genes with among-genotype differences clustered into eight modules. Modules 1, 3, and 7 were enriched for genes involved in digestion and proteolysis, cuticle development, and detection of chemical and biotic stimuli, respectively (Table S13). Module 8 was enriched for genes with very broad roles in cellular and metabolic processes, as well as reproduction. The remaining modules were not enriched for genes in any particular biological process, nor were any of the six modules calculated for genes significant among genotype and sex. Interestingly, one-quarter to one-third of these genes were nonprotein-coding (Figure 3E).

Thirty-five of the nonprotein-coding genes were correlated with total rRNA abundance, which indicates a stochastic or technical origin. Furthermore, 138 of the microenvironmental plasticity genes were potentially impacted by *Wolbachia* infection status (Table S6), and 62 by residual heterozygosity (Table S7). Unlike the genes differentially expressed among individuals, microenvironmental plasticity genes do not group into many over-represented biological process categories. Incomplete functional annotation is one potential reason for this, but an alternative explanation is that plasticity in gene expression is partially influenced by stochastic processes, as the very low broad-sense heritabilities for CV_E_ imply. Thus, while social interactions and individual responses to the environment shape the expression of some genes, stochastic processes influence others.

### Interactions between genes and replicate environment

**Figure 4:**
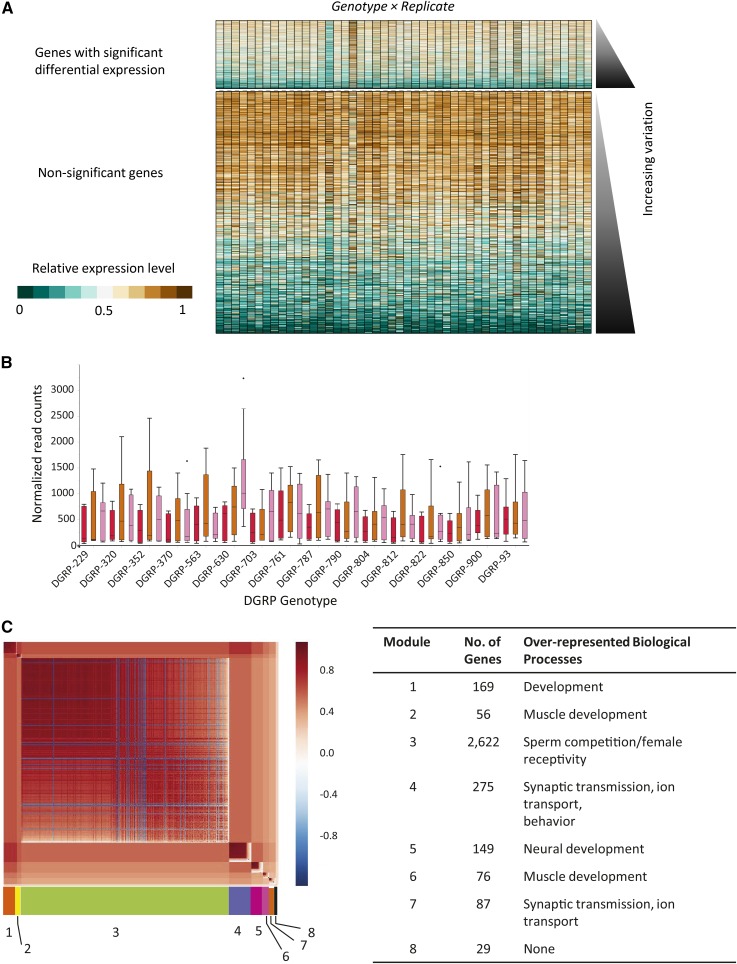
Genes with significant genotype-by-replicate environment interactions. (A) Heat map showing the relative expression levels (defined in Figure 2) of every detected gene in the genome by genotype and replicate. Genes are ordered from lowest to highest variability. (B) Representative boxplot showing the variation among genotype and replicate in *Shaker*. Box colors as in Figure 2. (C) MMC modules for genes with significant genotype-by-replicate environment interactions. The color scale and cluster orientation are as in Figure 2.

A classic debate in biology is whether genetic or environmental influences have a greater impact on organismal phenotypes. Considerable evidence suggests that genes may interact with the environment (reviewed in Scheiner 1993; for examples, see Han *et al.* 2011; Mahlios *et al.* 2013), and that the interaction can manifest itself in effects on gene expression (Idaghdour *et al.* 2010; Buil *et al.* 2015). Despite the restrictions we implemented to control replicate environments, we detected the differential expression of 3463 (22%) genes across genotype and replicate environment (Table 1, *G* × *R*). These differences were exemplified by gene expression of *Shaker* (Figure 4, A and B), a gene encoding a potassium ion channel required for neurotransmission (Salkoff *et al.* 1992) that has functions in sleep (Cirelli *et al.* 2005). To determine if genes with significant genotype-by-replicate-environment interactions might have features in common, we again used MMC and GO analysis (Bonferroni-corrected *P* < 0.05) (Figure 4C). This analysis grouped variably expressed genes into eight coexpressed modules (Table S14 and Table S15). Modules 1, 2, 5, and 6 were enriched for genes involved in developmental processes, with many developmental processes over-represented in Module 1, muscle development in Modules 2 and 6, and neural development in Module 5. Genes involved in behavioral responses to the environment such as reproductive behavior, phototransduction, learning and memory, and chemotaxis were enriched in Modules 3, 4, and 5, respectively. Modules 4 and 7 were enriched for cell communication, synaptic transmission, and ion transport. Module 8 was not enriched for any biological process. Thus, genes involved in mediating the response to environmental stimuli responded to fluctuations in each replicate environment.

### Genetic differences in transcript abundance

**Figure 5:**
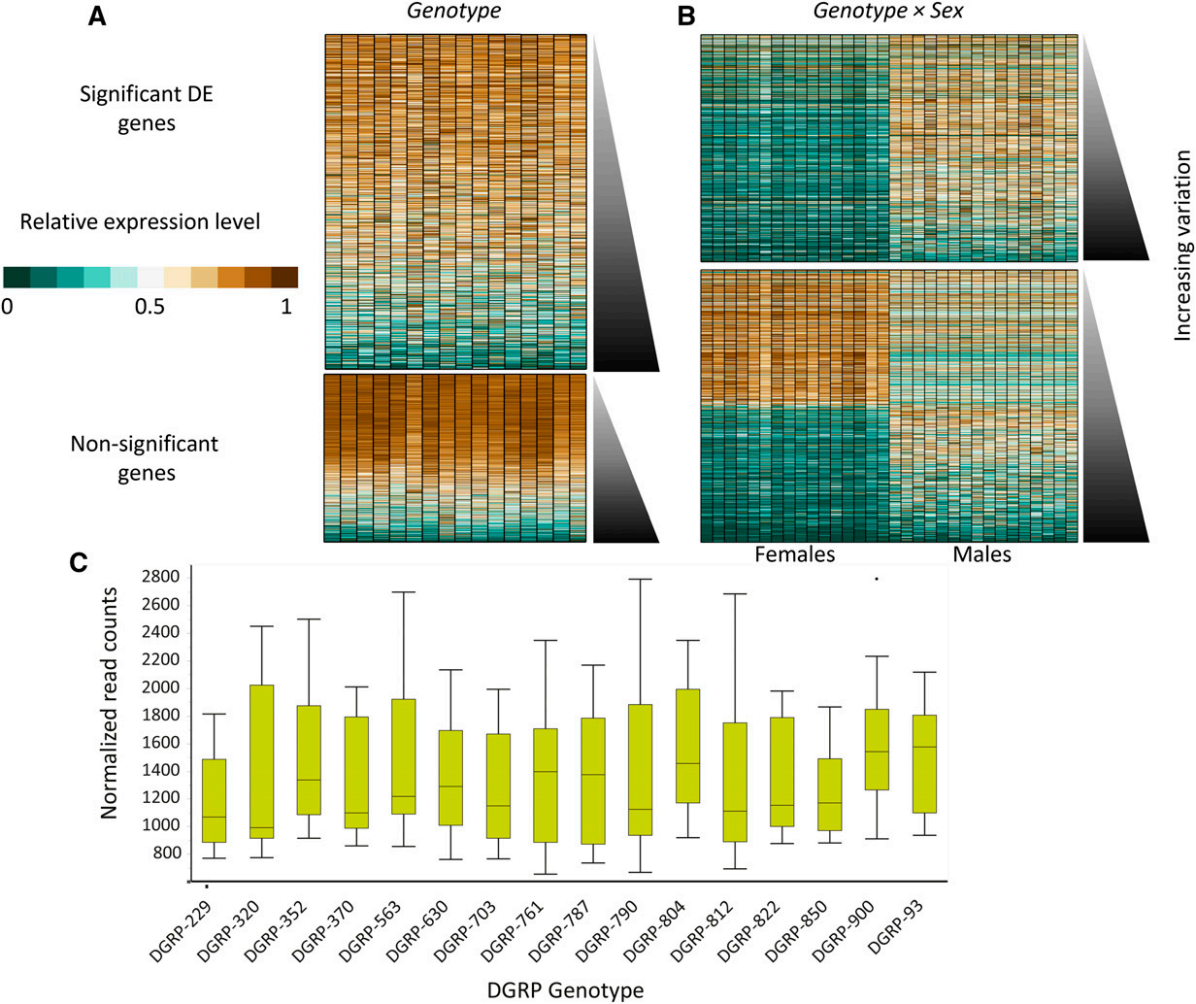
Genes differentially expressed across genotypes. Heat map showing the relative expression levels (defined in Figure 2) of every detected gene in the genome by (A) genotype and (B) genotype-by-sex. The orientation of the genes as in Figure 2. (C) Representative box plot showing the variation among genotype in *Phosphoglucose isomerase*.

We expected that genotype, with multiple differences in gene regulators, enhancers, and silencers, would profoundly influence gene expression, as demonstrated previously in pools of individual flies (Jin *et al.* 2001; Harbison *et al.* 2005; Wayne *et al.* 2007; Ayroles *et al.* 2009; Huang *et al.* 2015). Indeed, most of the 15,674 annotated transcripts we detected were differentially expressed among the DGRP genotypes (Table 1 and Table S1); 10,401 (66.4% of detected genes; FDR < 0.05) of the transcripts had genetic differences, and 7148 (45.6%) of genetically variable transcripts were also expressed in a sexually dimorphic manner. To determine whether variability was related to expression levels, we ordered the mean expression for each genotype by increasing variability, which revealed that highly expressed genes showed less variable expression, and genes with low expression were more variable (Figure 5A); the pattern was similar for the sexually dimorphic transcripts (Figure 5B). For many of these genes (5755), the difference between the lowest- and highest-expressed genotype was twofold or greater, but among the remaining genes (4646), the differences among lines were more subtle. Each genotype had variably expressed genes with both high and low gene expression, indicating that there was not a general effect on batteries of genes within a particular genotype or sex (Figure 5, A and B).

Variability can be observed in representative box plots showing the differences across DGRP genotypes for the gene *Calreticulin* (Figure 5C), which affects olfaction, startle response, and sleep (Stoltzfus *et al.* 2003; Sambandan *et al.* 2006; Harbison and Sehgal 2008; Yamamoto *et al.* 2008). Our results, which compare gene expression differences among individual flies, strongly support previous work demonstrating that sex and genotype are major contributors to expression differences.

### Latent plasticity in transcript abundance across replicate environments

**Figure 6:**
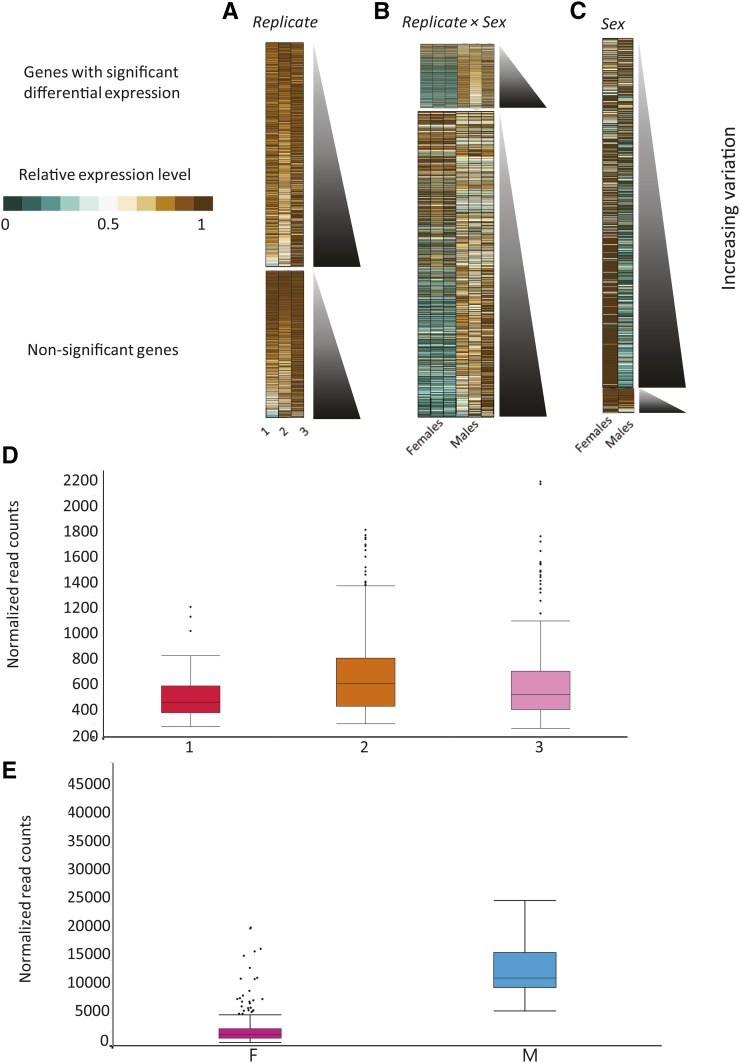
Genes differentially expressed among replicates and sexes. Heat map showing the relative expression levels (defined in Figure 2) of every detected gene in the genome by (A) replicate, (B) replicate-by-sex, and (C) sex. The orientation of the genes as in Figure 2. (D) Representative box plot showing the variation among environments in *Tenascin major*. Box colors as in Figure 2. (E) Representative box plot showing the variation among sexes in *sallimus*.

The experimental conditions were carefully controlled across replicates (see *Materials and Methods*); so gene expression changes among replicates should theoretically be zero. However, most of the detected transcripts were differentially expressed among replicate environments, indicating hidden or latent plasticity in gene expression; 9607 (61.3%, FDR < 0.05) genes had differentially expressed transcripts (Table 1 and Table S1). Thus, 61.3% of the transcriptome was sensitive to subtle environmental differences among identically reared cultures, while the remaining 38.7% of transcripts were robust to these fluctuations; 2646 (16.9%) were also sexually dimorphic in expression (Table 1). Transcriptional differences among replicates, like those across genotypes, were more subtle than those among individuals. Only 634 of the genes had differences that were twofold or greater, and many of the twofold or greater differences (325) were due to the average gene expression in one replicate being near zero. Unlike the differential expression across genotypes, there was little relationship among the magnitude of expression and the variability; highly expressed genes were equally likely to have high or low levels of variability (Figure 6, A and B). An example of differences across replicates can be observed in a plot of gene expression in *Tenascin-major* (*Ten-m*) (Figure 6D)—a gene with roles in eye morphogenesis, photoreceptor development, and synaptic growth (Kinel-Tahan *et al.* 2007; Mosca *et al.* 2012). The differences observed in *Ten-m* and the other differentially expressed genes across replicates suggest that latent plasticity in the transcriptome may exist whether environmental conditions are systematically altered or not.

### Sexual dimorphism in the transcriptome

As expected from previous studies of gene expression using pools of individuals, we observed massive differences in gene expression due to sex (Table 1 and Table S1) (Jin *et al.* 2001; Arbeitman *et al.* 2002; Parisi *et al.* 2003; Ranz *et al.* 2003; Harbison *et al.* 2005; Wayne *et al.* 2007; Zhang *et al.* 2007; Ayroles *et al.* 2009; Huylmans and Parsch 2014; Huang *et al.* 2015). Of the 15,674 genes detected in the experiment, 14,883, or 95%, were differentially expressed between males and females (FDR < 0.05). Many of these differences are likely to be due to differences in gene expression between reproductive tissues (Parisi *et al.* 2003). And 75% of genes with sex-biased expression had twofold or greater expression differences between males and females. We observed more male-biased (10,208 genes) than female-biased expression (Figure 6C). Some genes were nearly sex-specific in expression; 5447 of the transcripts that were differentially expressed between males and females had read counts below the minimal threshold of expression in females on average, suggesting male-specific expression, while 2194 genes had read counts below the minimum in males on average, indicating female-specific expression. Interestingly, however, variability in gene expression was higher in females than in males (Figure S1). Strong sex dimorphism can be seen, for example, in the gene *sallimus* (Figure 6E)—a gene with functions in muscle development and locomotion (Hakeda *et al.* 2000). Expression levels were much higher in males than in females for this gene. In summary, virtually every gene in the genome had significant differential expression between males and females.

## Discussion

Here we have shown that differences in gene expression among genetically identical individuals originate from responses to microenvironmental perturbations, and from stochastic factors originating at the cellular level. We found that 23% of the transcripts from individual flies of identical genotype reared in a common environment have differential expression. Gene expression differences among genetically identical individuals were quite common, and occurred despite the control of experimental conditions that affect gene expression, such as temperature, humidity, light:dark cycle, mating status, and social exposure. Why would there be so many genes differentially expressed among genetically identical individuals? One possibility may be differences in tissue size among individuals. For example, we observed differences in large numbers of reproduction-related genes, which suggests that there may be individual differences in reproductive tissue sizes among flies of a given sex and genotype. Tissue-specific differences could be mitigated in future experiments using body size or weight as a covariate. However, many of the genes we detected were involved in the behavioral response to external stimuli not known to vary with body size. These genes are aligned with adaptive responses as they have known functions in many aspects of behavior, such as locomotion, courtship, chemosensation, and sleep. This observation supports an adaptive *vs.* a stochastic model of gene expression control. In contrast, microenvironmental plasticity as measured by the coefficient of environmental variation (CV_E_) exhibits both adaptive and stochastic characteristics. Importantly, microenvironmental plasticity was influenced by genotype, and in some cases influenced by both genotype and sex. Genes with differential CV_E_ did not share a coherent pattern of biological function, which indicates that there may be subtle stochastic factors that influence expression. Alternatively, the fact that these genes are under genetic control and could be clustered together by expression variation implies that there may be functionally relevant biological processes that have not yet been thoroughly annotated. A third possibility is that genes with differential CV_E_ values may be linked to individual differences that are likely present, but that we did not control for, such as amount and timing of food intake, infection status, and larval density, though the number of genes falling into ontology categories known to impact these uncontrolled differences was low. Interestingly, we noted that some of these genes (27.6%) were nonprotein-coding RNAs, which are under increasing scrutiny as subtle regulators of protein-coding gene expression. In support of this idea, variation in nonprotein-coding RNA expression is observed among single cells (Shalek *et al.* 2013). In addition, cell-to-cell variability seems to be driven by external factors that are more global in nature (Raser and O’Shea 2005; Chang *et al.* 2008), as we also observed in individual flies. We suggest that the variability in expression of a subset of genes is an individualized response to subtle environmental differences, such as social organization or infection, and is under genetic control; the remaining variability is due to stochastic influences.

Our results indicate that two-thirds of the transcriptome varied among replicates, thus exhibiting phenotypic plasticity. This result differs from previous experiments (Sambandan *et al.* 2008; Zhou *et al.* 2012) measuring differential gene expression among applied environmental conditions in subsets of the DGRP. Only 20 genes changed in response to three different diets fed to larvae, despite the profound effects these diets had on olfactory avoidance behavior in the resulting adult flies (Sambandan *et al.* 2008). Furthermore, just 15% of the transcriptome was phenotypically plastic when 19 different environmental conditions were compared to a control environment (Zhou *et al.* 2012). The environmental conditions included strong treatments such as starvation, heat shock, and chill coma, and drugs such as fluoxetine, menadione, and nicotine (Zhou *et al.* 2012). This phenomenon is not confined to DGRP genotypes, as low numbers of transcripts responded to drug, temperature, and heavy metal treatments in other genotypes as well (Brown *et al.* 2014). The lower numbers of differentially expressed genes may be due to dissimilarities in experimental design and statistical power between these experiments and ours. A more intriguing possibility is that strong applied environmental treatments may activate gene expression in a few key pathways relative to the control environment, while, among untreated animals compared in replicate environmental conditions, the ability to perturb gene expression is maintained in a labile state, ready to respond to strong environmental changes should they arise. The relatively low magnitude differences we observed among replicates support this notion. Thus, gene expression exhibits canalization (Waddington 1959) only under strong environmental conditions.

When we rank order gene expression by variance in the differentially expressed genes in each factor category, we observed that genes with lower expression were among the most variably expressed due to genotype. Thus, within a genotype, noise abatement may be variable. However, there was no relationship between expression level and variance due to sex or replicate. It is well established that there are genes with highly sex-biased expression, but the variability in sex-biased responses within a sex has not been well studied, as pools of flies have usually been analyzed. We found greater expression variance in females. These results indicate that gene expression in females is either more responsive to random environmental and genotypic fluctuations than in males, if expression variance is adaptive, or that female gene expression is less robust than that of males. Given that females tend to be more resistant to many stresses (Matzkin *et al.* 2007; Mackay *et al.* 2012; Weber *et al.* 2012), there may be greater adaptive expression in females, even as expression in males is more variable across species, particularly for genes with male-biased expression (Zhang *et al.* 2007). Thus, evolution would appear to favor divergence in male gene expression between species, and more uniformity in male expression within species. Alternatively, differential variability between the sexes within a *D. melanogaster* may reflect differences in the relative contributions of additive *vs.* nonadditive or epistatic variance in males and females, which in turn affect the speed with which sexually dimorphic genes can adapt (Wayne *et al.* 2007).

Lack of “reproducibility” is often equated with error, but biological materials are inherently variable. Although low-level gene expression is difficult to accurately measure due to the sampling inherent in RNA-Seq measurements (McIntyre *et al.* 2011), we had superior statistical power to detect even low magnitude variance in gene expression (Lin *et al.* 2016). In addition, we applied stringent low-expression cutoffs based on an evaluation of expression in intergenic regions (Zhang *et al.* 2010; Lin *et al.* 2016). Duplicate libraries prepared for 118 flies indicated that the technical effects were very small relative to the biological effects (Lin *et al.* 2016). In addition, residual heterozygosity in chromosomal inversions does not appear to be a factor, in agreement with a recent assessment of gene expression in pooled flies of the DGRP (Huang *et al.* 2015). Thus, the biological sources of gene expression differences among individuals are much greater than these potential technical sources.

These findings have implications for our understanding of complex traits and disease. To the extent that gene expression influences quantitative traits, interindividual variability in gene expression in identical individuals interferes with our ability to link genotype with phenotype. While environmental factors specific to an individual might contribute to complex trait variation and the etiology of disease, efforts to find gene expression-based biomarkers might prove more successful if they were focused on genes that are robust across environments and over time.

## Supplementary Material

Supplemental Material
