# Supplementary material for: Microenvironmental Gene Expression Plasticity Among Individual Drosophila melanogaster
Source: G3 (Bethesda). 2016 Oct 20;6(12):4197–210. doi: 10.1534/g3.116.035444 (PMC5144987; doi:10.1534/g3.116.035444)
Supplement: Supplemental Material [file supp_6_12_4197__index.html]

Microenvironmental Gene Expression Plasticity Among Individual Drosophila melanogaster — Supplemental Material 

# Microenvironmental Gene Expression Plasticity Among Individual *Drosophila melanogaster*

## Supplemental Material for Lin *et al.*, 2016

**Files in this Data Supplement:**

- Figure S1 - Volcano plots of the ratio of (A) DESEq-normalized read counts, (B) dispersion (as computed by DESeq), and (C) CVE of DESeq-normalized read counts. (.pdf, 204 KB)
- Figure S2 - Heat maps showing relative dispersion for the CVE of each gene having a significant (A) Genotype and (B) Genotype?Sexcomponent. (.pdf, 192 KB)
- Table S1 - For each of the 15,674 genes for which expression was detected, the table lists feature type (protein coding, non-protein coding, etc.), chromosome arm location, gene symbol, gene name, mean DESeq-normalized read counts for each genotype/sex/replicate, *P*-value/False Discovery Rate (FDR) for each factor analyzed in the experiment, and broad-sense heritability (H2) estimate. (.xlsx, 22 MB)
- Table S2 - Numbers of genes differentially expressed at different false discovery rates (FDR). (.xlsx, 13 KB)
- Table S3 - Comparison of differentially expressed genes called by DESeq with limma-voom with quality weights. (.xlsx, 13 KB)
- Table S4 - Comparison of original model with a model where plate and muliplex pool effects were added as covariates. (.xlsx, 13 KB)
- Table S5 - Results of Brown-Forsythe and Levene's heterogeneity of variance tests showing that gene expression is variable among individuals, i.e., within a genotype/replicate/sex condition. (.xlsx, 708 KB)
- Table S6 - Test of significant differences in gene expression according to *Wolbachia pipiensis* infection status. (.xlsx, 498 KB)
- Table S7 - Correlation between the standard deviation of normalized read counts for each DGRP line and the percentage heterozygosity per line. (.xlsx, 496 KB)
- Table S8 - Modulated Modularity Clustering results for genes differentially expressed among individual flies. (.xlsx, 350 KB)
- Table S9 - Gene ontology biological process categories over-represented in modules of differentially expressed genes among individual flies. (.xlsx, 21 KB)
- Table S10 - For each of the 15,674 genes for which expression was detected, the table lists feature type (protein coding, non-protein coding, etc.), chromosome arm location, gene symbol, gene name, the coefficient of environmental variation (CVE) for each genotype/sex/replicate, the *P*-value/False Discovery Rate (FDR) for each factor analyzed in the experiment, and broad-sense heritability (H2) estimate. (.xlsx, 20 MB)
- Table S11 - Modulated Modularity Clustering results for genes with significant micro-environmental plasticity (as measured by CVE) among genotypes. (.xlsx, 118 KB)
- Table S12 - Modulated Modularity Clustering results for genes with significant micro-environmental plasticity (as measured by CVE) among genotype and sex. (.xlsx, 36 KB)
- Table S13 - Gene ontology biological process categories over-represented in modules for micro-environmental plasticity (measured by CVE) by genotype. (.xlsx, 14 KB)
- Table S14 - Modulated Modularity Clustering results for genes with significant genotype-by-replicate interactions. (.xlsx, 323 KB)
- Table S15 - Gene ontology biological process categories over-represented in modules of differentially expressed genes having genotype-by-replicate interactions. (.xlsx, 21 KB)
