# Supplementary material for: Microenvironmental Gene Expression Plasticity Among Individual Drosophila melanogaster
Source: G3 (Bethesda). 2016 Oct 20;6(12):4197–210. doi: 10.1534/g3.116.035444 (PMC5144987; doi:10.1534/g3.116.035444)

Supplemental Figure S1. Volcano plots of the ratio of (A) DESEq-normalized read counts, (B) dispersion (as computed by DESeq), and (C) CV_E of DESeq-normalized read counts.

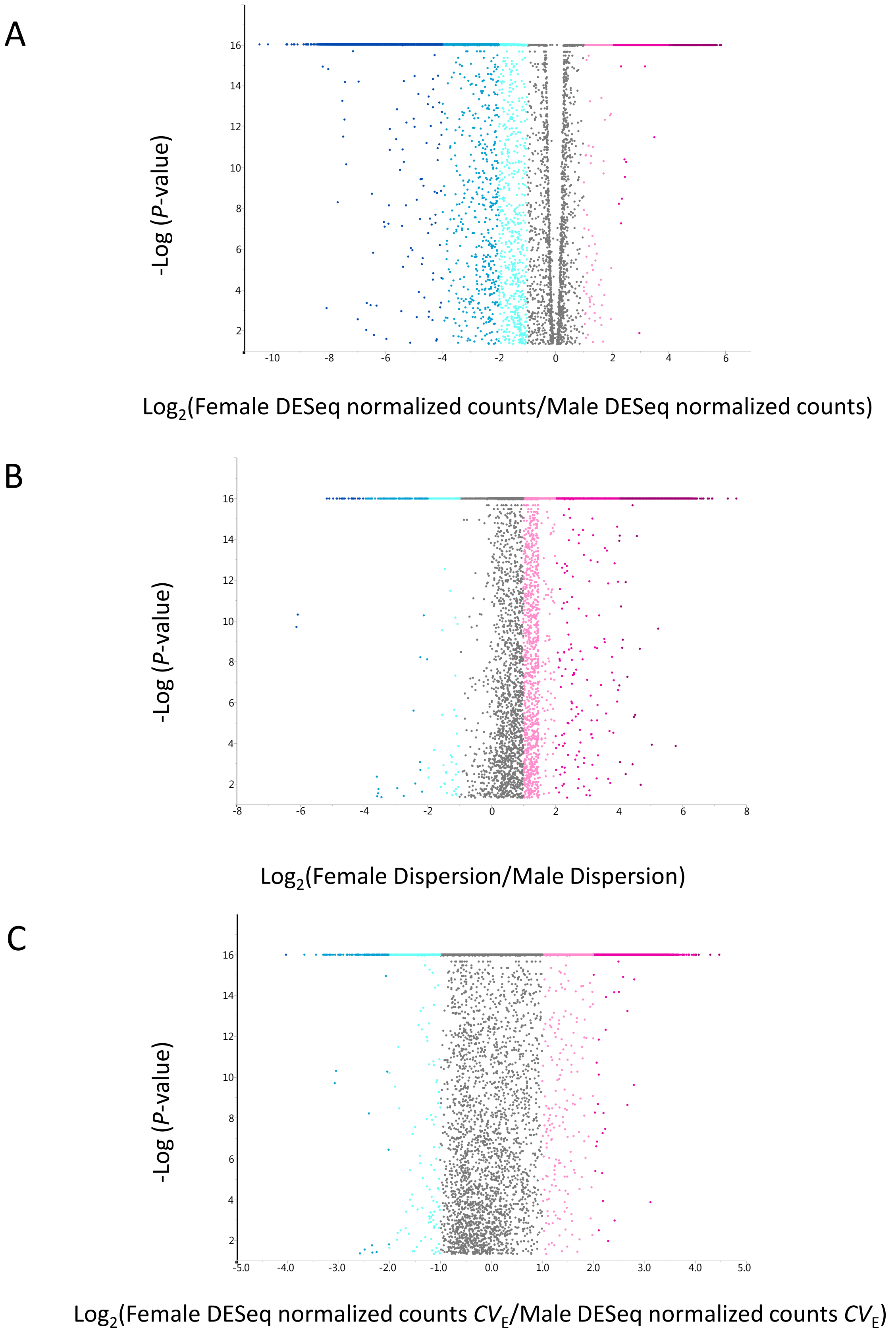

Supplement: Supplemental Material [file supp_g3.116.035444_FigureS1.pdf]
