# Supplementary material for: Microenvironmental Gene Expression Plasticity Among Individual Drosophila melanogaster
Source: G3 (Bethesda). 2016 Oct 20;6(12):4197–210. doi: 10.1534/g3.116.035444 (PMC5144987; doi:10.1534/g3.116.035444)

Supplemental Figure S2. Heat maps showing relative dispersion for the CV_E of each gene having a significant (A) Genotype and (B) Genotype×Sex component. CVE is related to expression mean (μ) and dispersion (ϕ) by the equation $CV_E = \mathrm{sqrt}(1/\mu + \phi)$. The relative dispersion is obtained by dividing the average dispersion for each line by the maximum dispersion among all lines.

A. Genotype

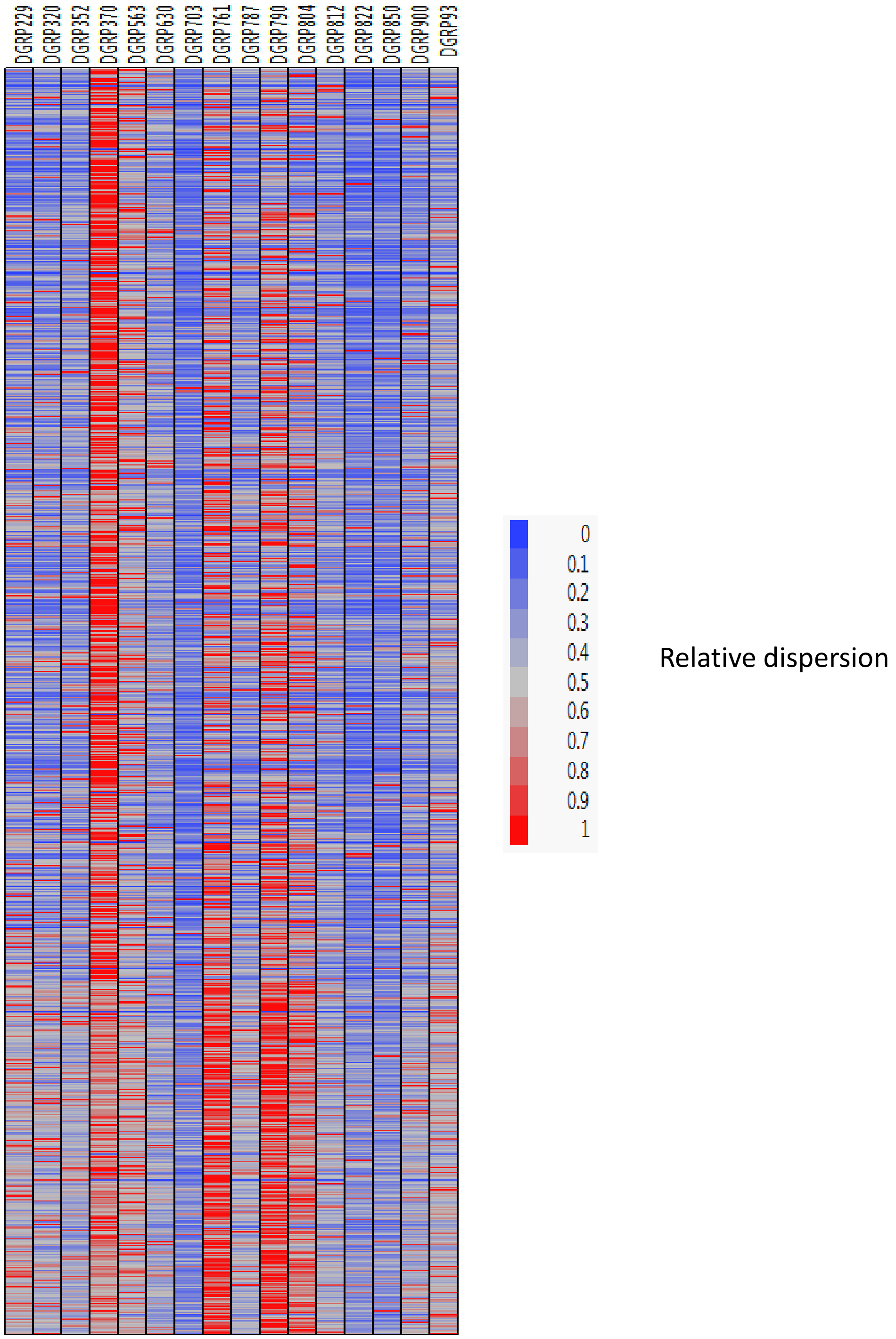

B. Genotype×Sex

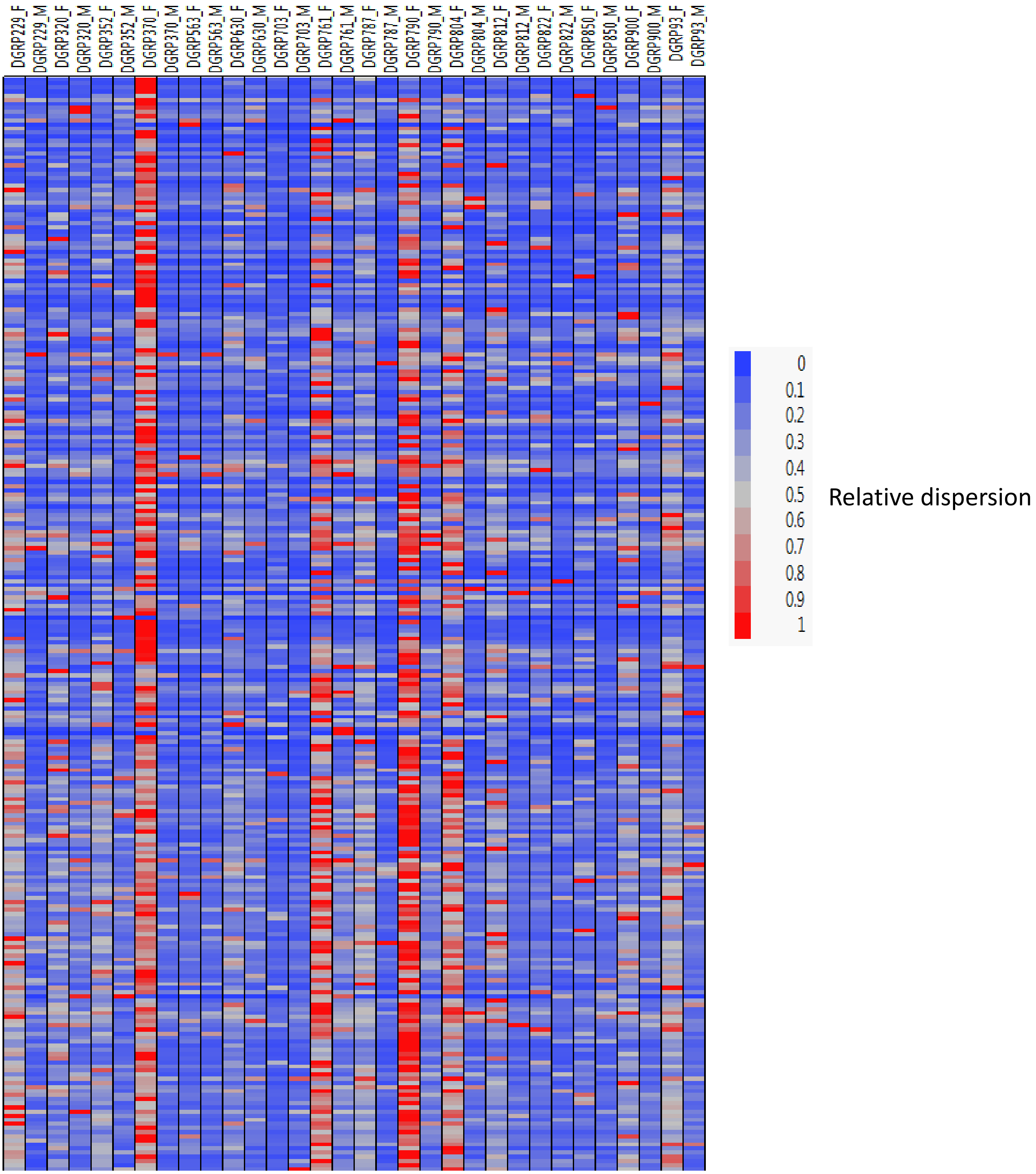

Supplement: Supplemental Material [file supp_g3.116.035444_FigureS2.pdf]
